# Cajanol Sensitizes A2780/Taxol Cells to Paclitaxel by Inhibiting the PI3K/Akt/NF-κB Signaling Pathway

**DOI:** 10.3389/fphar.2021.783317

**Published:** 2021-12-08

**Authors:** Ming Sui, Hairong Yang, Mingqi Guo, Wenle Li, Zheng Gong, Jing Jiang, Peiling Li

**Affiliations:** ^1^ Department of Obstetrics and Gynecology, Second Affiliated Hospital of Harbin Medical University, Harbin, China; ^2^ Department of Obstetrics and Gynecology, The Second Affiliated Hospital of Fujian Medical University, Quanzhou, China; ^3^ Department of Obstetrics and Gynecology, First Hospital of Qiqihar, Qiqihar, China

**Keywords:** cajanol, MDR, paclitaxel resistance ovarian cancer, P-glycoprotein, PI3K/AKT/NF-κB pathway

## Abstract

Ovarian cancer is the second most common gynecological malignancy, and one of the most deadly. The bottleneck restricting the treatment of ovarian cancer is its multi-drug resistance to chemotherapy. Cajanol is an isoflavone from pigeon pea (Cajanus cajan) that has been reported to have anti-tumor activity. In this work, we evaluate the effect of cajanol in reversing paclitaxel resistance of the A2780/Taxol ovarian cancer cell line *in vitro* and *in vivo*, and we discuss its mechanism of action. We found that 8 μM cajanol significantly restored the sensitivity of A2780/Taxol cells to paclitaxel, and *in vivo* experiments demonstrated that the combination of 0.5 mM/kg paclitaxel and 2 mM/kg cajanol significantly inhibited the growth of A2780/Taxol metastatic tumors in mice. Flow cytometry, fluorescence quantitative PCR, western blotting and immunohistochemical staining methods were used to study the mechanism of reversing paclitaxel resistance with cajanol. First, we determined that cajanol inhibits paclitaxel efflux in A2780/Taxol cells by down-regulating permeability glycoprotein (P-gp) expression, and further found that cajanol can inhibit P-gp transcription and translation through the PI3K/Akt/NF-κB pathway. The results of this work are expected to provide a new candidate compound for the development of paclitaxel sensitizers.

## Introduction

Ovarian cancer is one of the three major malignant tumors of the female reproductive system and has the highest death rate among all the gynecological malignancies (Deb et al., 2018). Because ovarian cancer is difficult to diagnose, most ovarian cancer patients are recognized at an advanced stage, leading to a decreased survival rate (Grunewald and Ledermann, 2017). At present, the combination of paclitaxel and platinum chemotherapy is the primary regimen for ovarian cancer patients. At the beginning of the chemotherapy regimen, these drugs are effective for more than 80% of patients, but cancer cells develop resistance to the drugs, which results in cancer recurrence, leading to a five-year survival rate of 45% for ovarian cancer patients. Moreover, the survival rate of cancer patients diagnosed as terminal is less than 30% (Webb and Jordan, 2017).

Studies have shown that drug resistance to paclitaxel involves various mechanisms, including the increase of multi-drug resistance (MDR) proteins (Mihanfar et al., 2019), and alterations in expression of vascular endothelial growth factor (Akiyama et al., 2012), matrix metalloproteinases (Hu et al., 2012; Kato et al., 2012) or microtubule associated proteins (Ren et al., 2016). MDR occurs in many human cancers, including colon cancer, breast cancer and kidney cancer (Amawi et al., 2019) and causes resistance to chemotherapy. Overexpression of ABC transporters such as MDR1/P-gp is considered to be the classical mechanism of drug resistance (Waghray and Zhang, 2018). P-gp is encoded by the *ABCB1* gene, and the P-gp promoter sequence contains a κB site, which can be recognized and activated by nuclear factor kappa B (NF-κB) (Loaiza et al., 2016). There have been reports that the PI3K/Akt signal transduction pathway is involved in NF-κB-mediated MDR (Solt and May 2008). It has also been reported that ivermectin and ferulic acid can reverse drug resistance by inhibiting the EGFR/ERK/Akt/NF-κB pathway, down-regulating the expression of P-gp (Muthusamy et al., 2019). This suggests the use of suitable inhibitors to inhibit NF-κB-mediated P-gp overexpression as a feasible method to reverse MDR.

Natural products from plants are effective sources of anti-tumor drugs, and some have been shown to modulate MDR. Many types of natural products, such as flavonoids, alkaloids and terpenes, have been demonstrated to inhibit P-gp. Cajanol is an isoflavanone compound derived from the root of *C. cajan*, and has a variety of pharmacological actions, including antibacterial, antifungal, antimalarial and antitumor activities (Zhao et al., 2013). In this study we demonstrate for the first time the inhibition of P-gp by cajanol, using cell proliferation, rhodamine accumulation, fluorescence quantitative PCR, western blot, and *in vivo* assays. We investigated the effects of cajanol on the expression and function of P-gp, and we also examined the regulatory effects of cajanol through the PI3K/Akt/NF-κB signaling pathway to mediate P-gp proteins. The objective was to further develop cajanol for the reversal of ovarian cancer drug resistance and to provide data for the potential clinical application of cajanol.

## Materials and Methods

### Cell Lines, Antibodies and Reagents

The A2780 human ovarian cancer cell line and the A2780/Taxol paclitaxel-resistant ovarian cancer cell line were purchased from Keygen Biotech (Jiangsu, China). The human non-small cell lung cancer cell line A549 and the paclitaxel-resistant cell line A549/Taxol were donated by Prof. Yang from Qiqihar Medical College. All the cells were cultured in RPMI-1640 cell culture medium (Solarbio Science and Technology Co., Ltd., Beijing, China) at 37°C and 5% CO_2_. The culture medium was supplemented with 10% inactivated fetal bovine serum (Gibco, NY, United States) and 1% penicillin and streptomycin. The A2780/Taxol cells were cultured in medium containing 800 ng/mL paclitaxel and then switched to a non-drug medium 2 weeks before the experiment. An antibody against P-gp (0.1 μg/m; ab170904), NF-κB/p65 (1/2000, ab32536), p-NF-κB/p65 (1/1000, ab239882) and VEGF (1/1000, ab32152) were purchased from Abcam (Cambridge, United Kingdom); antibodies against PI3K (1/1000, #4292), Akt (1/1000, #9272), *p*-Akt (1/1000, #9271), *α*-Tubulin (1/2000, #2144), MMP-9 (1/1000, #3852), MRP1 (1/1000, ab233383), MRP2 (1/2000, ab172630), LRP (1/1000, #64099), lamin B (1/1000, #17416) and *ß*-actin (1/1000, #4970) were purchased from Cell Signaling Technology (CST, MA, United States).

**FIGURE 1:**
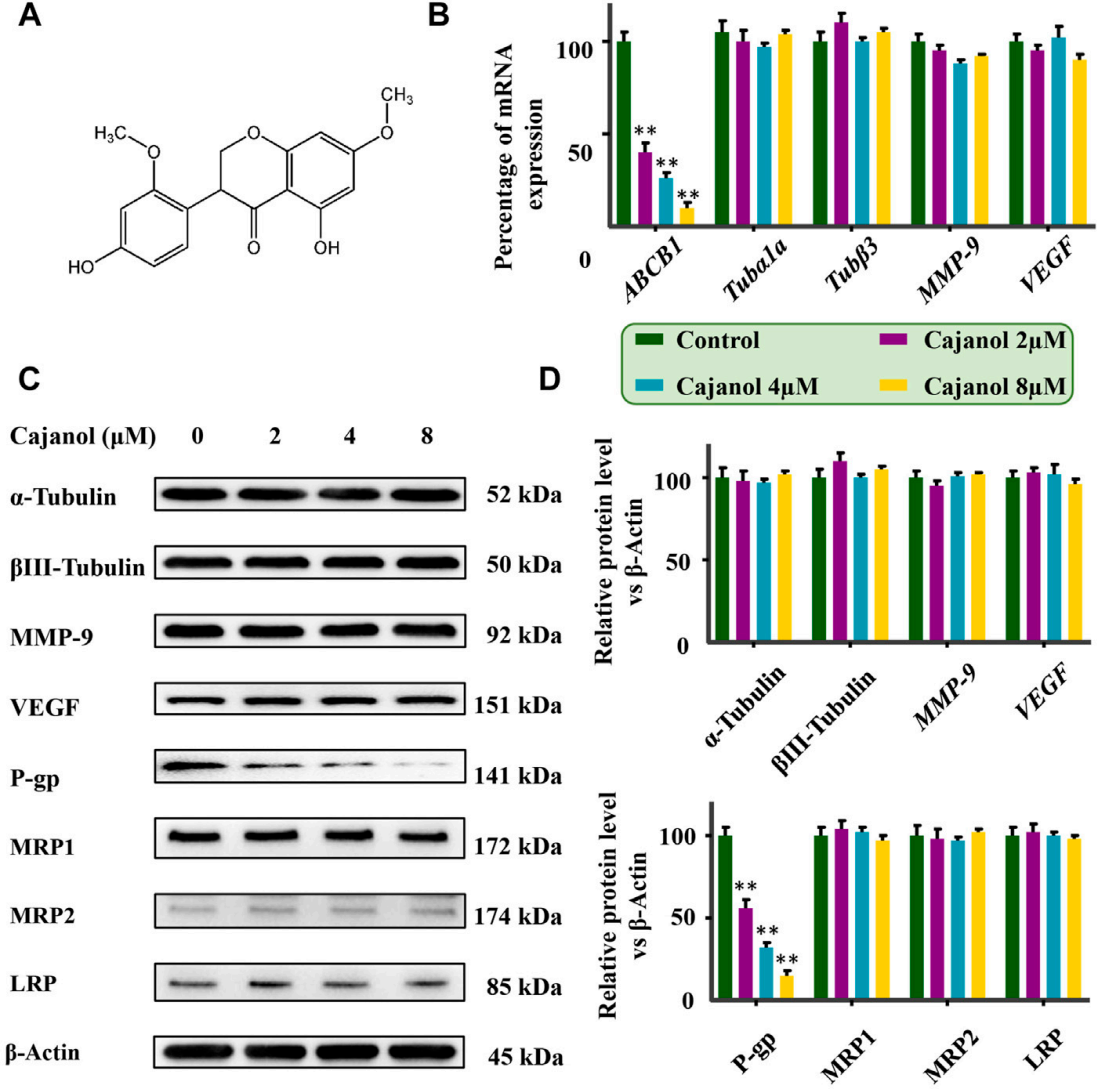
Effect of cajanol on ABCB1 mRNA and P-gp protein expression in ovarian cancer cells. Cells were treated with cajanol (0, 2, 4 or 8 μM) for 48 h. **(A)** Chemical formula of cajanol. **(B)** Effect of cajanol on *ABCB1*, *MMP-9*, *VEGF*, *Tubα1a* and *Tubβ3* mRNA expression in A2780/Taxol cells as determined by RT-qPCR assay. **(C)** Expression of α-tubulin, βIII-tubulin, VEGF, MMP-9, P-gp, MRP1, MRP2 and LRP protein in A2780/Taxol cells after treated with cajanol. **(D)** Expression was normalized with *ß*-actin, respectively. Each column shows the mean ± S.D. *p < 0.05, **p < 0.01 *vs*. control group.

Cajanol was extracted by our laboratory with purity greater than 98%. The structural formula is shown in Figure 1A. The extraction method of cajanol refers to the published extraction method with slightly modified (Liu et al., 2011). Specifically, the crushed pigeon pea root was impregnated with ethanol-water (80:20, V/V) solution for 24 h with a solid-liquid ratio of 1:10, repeated 3 times. The filtrate was concentrated using a rotary evaporator to obtain a crude extract. And the crude extract was extracted with ethyl acetate/distilled water (3/1, v/v), the ethyl acetate was separated and concentrated to obtain brown product. The brown product was separated by a resin column, water and ethanol were used as mobile phases, and the 50% ethanol eluted part was retained for further purification. Using a silica gel column with chloroform-methanol as the mobile phase, cajanol was separated from the chloroform-methanol (12:1, v/v) fraction. After crystallization and recrystallization, white crystals were obtained. The HPLC and negative ion mode mass spectra of Cajanol are shown in Supplementary Figure S1. All other reagents were purchased from Sigma (St. Louis, MO, United States).

### Methylthiazolyldiphenyl-Tetrazolium Bromide Assay

Cells to be tested (100 µL; 5 × 10^3^ cells/well) were seeded in a 96-well plate and cultured overnight. Paclitaxel and cajanol were prepared as stock solutions in dimethyl sulfoxide (DMSO) and diluted (1000, 500, 250, 125, 62.5, 31.25, 15.63, 7.81, 3.91, 1.96 and 0.98 μM). Seeded cells were treated in quadruplicate with the diluted solutions for 72 h. MTT was added, and the cells were incubated for 4 h. The culture medium was then removed, and DMSO (150 µL) was added. The plate was placed in a shaker to fully dissolve the purple formazan crystals. The OD was measured at 570 nm, and the growth inhibition rate was calculated. All the experiments were repeated in triplicate. GraphPad Prism 7.0 (GraphPad Software, Inc., La Jolla, CA, United States) was used to calculate the IC_50_ values. Fold resistance (FR) is calculated by dividing the IC_50_ obtained in the resistant cancer cells (with or without the reversal compounds) by the IC_50_ of the non-resistant, parental cancer cells (Hussein et al., 2017).

### qRT-PCR

Medium containing 2, 4 or 8 µM cajanol was prepared and incubated with A2780/Taxol cells to determine the effect of cajanol on *ABCB1*, *MMP-9*, *VEGF*, *Tubα1a* and *Tubβ3* mRNA expression. After 48 h of co-incubation, total RNA was isolated using a RNA Extraction Kit (Takara Bio Inc., Dalian, China), and total RNA reverse transcription was performed using a Reverse Transcription Kit (Takara). Real-time quantitative PCR primers were designed using Primer Premier 5.0 (Supplementary Table S1), and SYBR^®^ Premix Ex Taq™ II kit (Takara) was used for the RT-qPCR assay. *β-actin* was selected as the internal reference gene; the reaction conditions have been previously described (Huang et al., 2017). Relative gene expression was calculated by the 2^−ΔΔ Ct^ method.

### Rhodamine Accumulation

Logarithmic cells (4 × 10^5^/mL density) were seeded in a 12-well plate. Positive control verapamil (8 µM) and appropriate concentrations of cajanol were added, and the cells were incubated for 2 h at 37°C. Then, rhodamine-123 at a final concentration of (1 μg/mL) was added, and the cells were incubated at 37°C, 5% CO_2_ for 1 h. After the reaction was completed, the supernatant was discarded after centrifugation at 4°C, and the cells were washed twice with pre-chilled PBS buffer (4°C), terminating the rhodamine efflux. The cells were then re-suspended in cold PBS and the intracellular drug fluorescence was determined by flow cytometry (Beckman Coulter, Fullerton, CA, United States), with an excitation wavelength of 466 nm and an emission wavelength of 535 nm. The results were analyzed by Expo32ADC software (Beckman Coulter).

### Western Blot

To determine whether cajanol affects the expression of P-gp through the PI3K/Akt signaling pathway, we measured the expression of P-gp, t-Akt, *p*-Akt, NF-κB/p65 and p-NF-κB/p65 in A2780/Taxol cells. The A2780/Taxol cells were treated with cajanol (2, 4, or 8 μM) for 48 h before the total cell protein was extracted. Cell lysis buffer for Western and IP (P0013, Beyotime Biotechnology, Shanghai, China) is selected as the whole cell protein extraction lysate. The nucleoprotein separation method is strictly implemented in accordance with the instructions. Specifically, the cells are scraped off with a cell scraper, and the cell pellet is collected after centrifugation. Add 200 µl of cytoplasmic protein extraction reagent A supplemented with PMSF for every 20 µl of cell pellet. Vortex for 5 s to completely suspend and disperse the cell pellet. After ice bath for 10 min, add 10 µl of cytoplasmic protein extraction reagent B and Vortex for 5 s. After the sample was ice bathed for 1 min, Vortex again for 5 s, and then placed in a 4°C centrifuge at 12,000 g for 5 min. Immediately draw the supernatant into a pre-cooled plastic tube, which is the cytoplasmic protein extracted. Equal amounts of proteins were separated by SDS-PAGE and transferred to a polyvinylidene fluoride membrane. After blocking, the membrane-bound proteins were probed with the relevant primary antibodies. The membranes were washed and then incubated with the secondary antibodies; finally antibody-bound proteins were detected using enhanced chemiluminescence reagents.

### *In Vivo* Experiments

Six-week-old healthy female pure BABL/c nude mice (weighing 18–20 g) were purchased from Vital River Experimental Animal Co. Ltd. (Beijing, China). The mice were placed in a thermostatted laminar flow box and kept in a specific pathogen-free environment with a 12 h/12 h light/dark cycle. The animal experiments and use of tumor cells were approved by the Animal Care Welfare Committee of the First hospital of Qiqihar according to the ethical guidelines of the animals (scientific procedures) act 1986 amendment regulations (SI 2012/3039); Approval number: QAEC20190043.

A2780/Taxol cells were cultured in RPMI-1640 culture medium containing 10% fetal bovine serum. Logarithmic growth phase cells were collected to prepare a suspension with a concentration of living cells of 1×10^5^/mL. Live cells accounted for more than 95% of the population. The cell suspension was inoculated subcutaneously in the right armpit near the back of each nude mouse (approximately 10^5^ cells). After 1 week of inoculation, 16 mice with successful tumor formation were randomly divided into four groups, ensuring adequate numbers for statistical analysis: normal saline group, Taxol group (0.5 mM/kg), cajanol group (2 mM/kg) and paclitaxel (0.5 mM/kg) + cajanol (2 mM/kg) combined group. The agents were administered through the caudal vein on the 1st, 8th and 15th day after successful tumor implantation. The body weights and condition of the mice and growth of the transplanted tumors were observed every 3 days for 24 days. The tumor volume was calculated with the following formula: V = (length × width^2^)/2. When the tumor diameter was over 2 cm or the ulcer area was greater than 4 mm, the mice were euthanized by cervical dislocation after inducing a coma by inhalation of 2.5% isoflurane vapor. Remaining mice were euthanized after 24 days. The tumor tissue was collected for immunohistochemistry, fluorescence quantitative PCR and western blot analyses.

### Immunohistochemical Staining

Immunohistochemistry was performed on paraformaldehyde-fixed paraffin-embedded sections. The detailed staining procedure has been described previously (Xu et al., 2017).

### Statistical Analysis

Statistical analyses were carried out using GraphPad Prism 7.0 (GraphPad Software, La Jolla, CA). One-way ANOVA was performed between groups. For ANOVA, the observed variance was partitioned into components according to different explanatory variables. *p < 0.05 was considered to be significant.

## Results

### Cajanol was Able to Reverse the Resistance of A2780/Taxol and A549/Taxol Cells to Paclitaxel.

**FIGURE 2:**
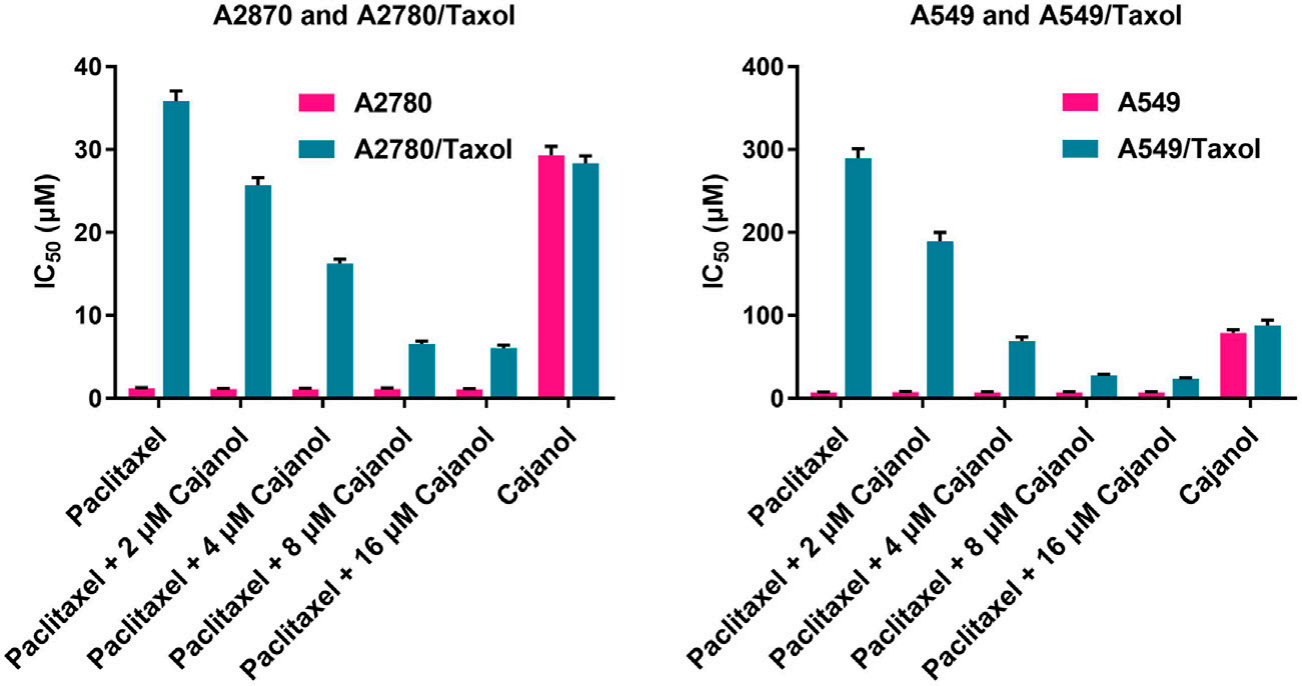
Effect of cajanol on intracellular Rhodamine-123 accumulation in A2780 and A2780/Taxol cells. Rhodamine-123 fluorescence intensity was measured using a flow cytometer and expressed as the mean ± SD. *p < 0.05, **p < 0.01 *vs.* A2780.

The paclitaxel MTT IC_50_ values after treatment of A2780 and A2780/Taxol cells for 72 h were 1.23 ± 0.10 µM and 35.85 ± 1.23 µM, respectively (Table 1and Figure 2). The IC_50_ values in A2780/Taxol cells for paclitaxel treatment combined with 2, 4, 8 or 16 μM cajanol were 25.67 ± 0.94 µM, 16.25 ± 0.54 µM, 6.54 ± 0.37 µM and 6.05 ± 0.33 µM, respectively. When cajanol concentration was 0 µM, 2 µM, 4 µM, 8 µM or 16 μM, the fold resistance of A2780/Taxol cells on A2780 cells were 29.15, 22.52, 14.51, 5.64 and 5.50, respectively. For A549/Taxol cells, cajanol also showed a good drug resistance reversal effect. The IC_50_ of paclitaxel on A549 cells and A549/Taxol cells were 7.52 ± 0.46 µM and 289.34 ± 11.46 µM, respectively. After treatment with 2 µM, 4 µM, 8 µM or 16 μM cajanol, the IC_50_ of paclitaxel for A549 cells was almost unchanged, but the IC_50_ for A549/Taxol cells decreased to 189.43 ± 10.87 µM, 68.95 ± 4.87 µM, 27.9 ± 1.23 µM and 23.76 ± 1.12 µM, respectively. And the corresponding fold resistance is also reduced from 38.48 to 3.15. The results showed that co-treatment of cajanol with paclitaxel substantially inhibits A2780/Taxol cells and that the IC_50_ value is decreased by more than 4-fold at a cajanol concentration of 8 µM. These results suggest that cajanol effectively reverses the resistance of A2780/Taxol cells to paclitaxel and that the effect is concentration dependent at levels up to 8 µM.

**TABLE 1:** Effect of cajanol on the cytotoxicity of paclitaxel in A2780/Taxol and A2780 cells by MTT assay.

Drug and concentrations	IC_50_ (µM)					
	A2780	A2780/Taxol	FR	A549	A549/Taxol	FR
Paclitaxel	1.23 ± 0.10	35.85 ± 1.23	29.15	7.52 ± 0.46	289.34 ± 11.46	38.48
Paclitaxel + 2 µM Cajanol	1.14 ± 0.09	25.67 ± 0.94	22.52	7.78 ± 0.56	189.43 ± 10.87	24.35
Paclitaxel + 4 µM Cajanol	1.12 ± 0.12	16.25 ± 0.54	14.51	7.65 ± 0.47	68.95 ± 4.87	9.01
Paclitaxel + 8 µM Cajanol	1.16 ± 0.13	6.54 ± 0.37	5.64	7.46 ± 0.60	27.9 ± 1.23	3.74
Paclitaxel + 16 µM Cajanol	1.10 ± 0.09	6.05 ± 0.33	5.50	7.55 ± 0.49	23.76 ± 1.12	3.15
Cajanol	29.33 ± 1.04	28.34 ± 0.90	0.97	78.89 ± 3.87	87.78 ± 6.87	1.11

a Fold resistance (FR) is calculated by dividing the IC_50_ obtained in the resistant cancer cells (with or without the reversal compounds) by the IC_50_ of the non-resistant, parental cancer cells.

### Cajanol Restores the Sensitivity of Paclitaxel to A2780/Taxol Cells by Inhibiting the Expression of P-gp Protein

In order to study the mechanism by which cajanol restores the sensitivity to paclitaxel of A2780/Taxol cells, the expression of resistance-related genes in A2780/Taxol cells was determined after treatment with different concentrations of cajanol (2, 4 or 8 μM). The expression of ABCB1 gene was significantly down-regulated by treatment with cajanol in a concentration-dependent manner (Figure 1B). When the concentration of cajanol reached 8 μM, the expression of ABCB1 gene was about 10% of the control group. The expression levels of VEGF, MMP-9, Tubα1a and Tubβ3 did not change. Further, western blots were used to determine the protein expressions of P-gp, VEGF, MMP-9, α-Tubulin and βIII-Tubulin in A2780/Taxol cells after cajanol treatment, and the results were consistent with the results of fluorescence quantitative PCR (Figures 1C,D). The expression levels of VEGF, MMP-9, α-Tubulin and βIII-Tubulin were not significantly changed, while expression levels of P-gp were significantly reduced. These results suggest that cajanol can reverse paclitaxel sensitivity by inhibiting this MDR protein in A2780/Taxol cells.

In order to further determine whether cajanol has the same inhibitory effect on other MDR proteins, we determined the expression of MRP1, MRP2 and LRP proteins in A2780/Taxol cells treated with cajanol. Western blot results showed that the expression levels of MRP1, MRP2 and LRP were not significantly changed (Figures 1C,D). These results all indicate that cajanol specifically reduces expression of ABCB1 and P-gp.

### Cajanol Inhibits the Transport of P-gp Protein in A2780/Taxol Cells

**FIGURE 3:**
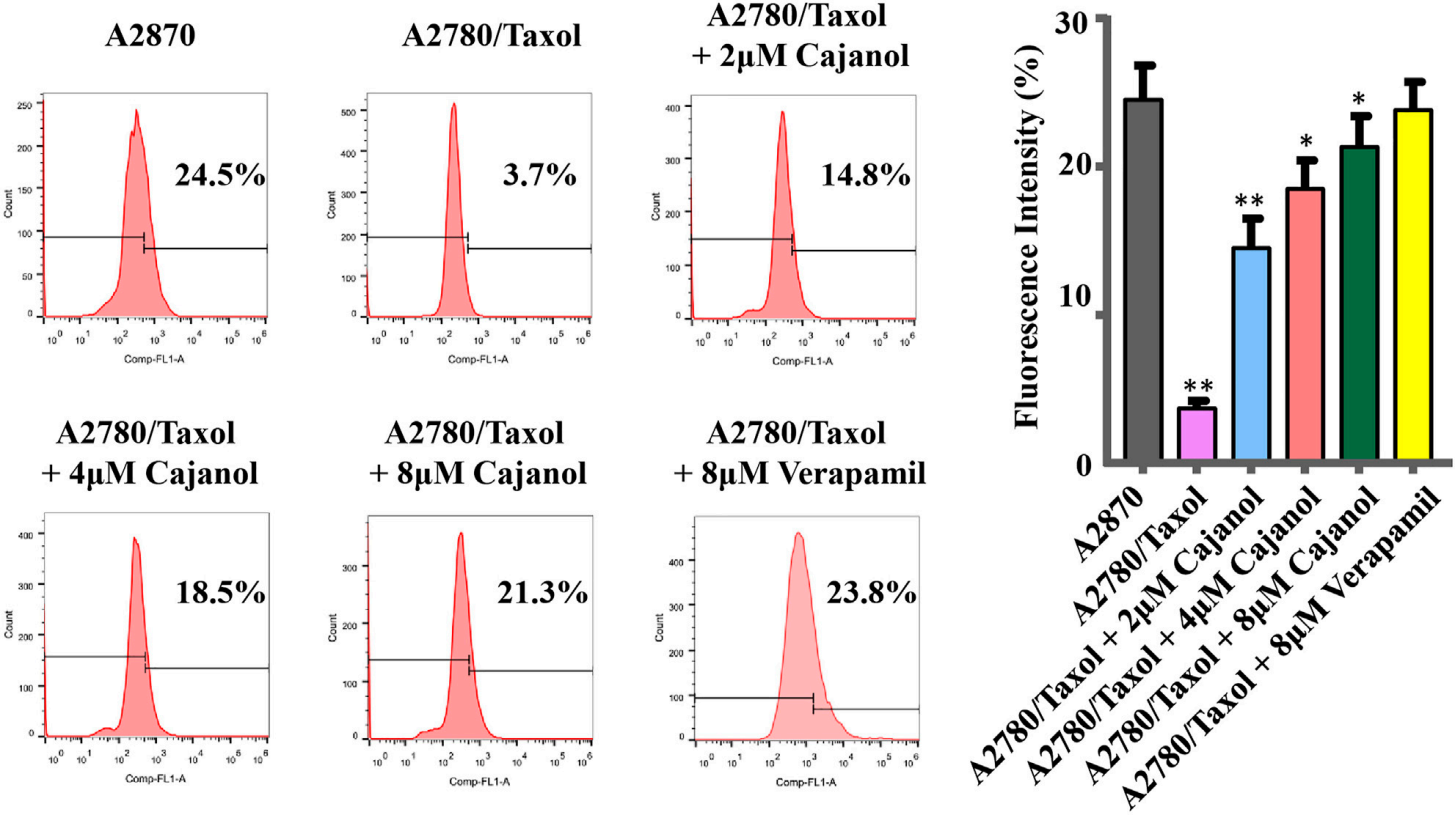
Cajanol treatment down-regulates P-gp expression via inhibition of the PI3K/Akt/NF-κB signaling pathway. Cajanol inhibits the phosphorylation of IκB and the translocation of NF-κB from the cytoplasm to the nucleus by inhibiting the expression of PI3K and the phosphorylation of Akt, and ultimately inhibits the transcription and translation of the P-gp protein.

Flow cytometry was used to assess the effect of cajanol on P-gp protein transport. Rhodamine-123 is transported by P-gp and can be detected by its emission of yellow-green fluorescence. By monitoring Rhodamine-123 accumulation in cells, we evaluated the transport capacity of P-gp protein in A2780/Taxol cells treated with cajanol at different concentrations (Figure 3). The intracellular fluorescence intensity of control cells A2780 (24.5%) was found to be significantly higher than that of untreated A2780/Taxol cells (3.7%). After treatment with 2, 4 or 8 μM cajanol, the fluorescence intensity in A2780/Taxol cells increased to 14.8, 18.5 and 21.3% respectively. The P-gp inhibitor verapamil also increased the fluorescence intensity of A2780/Taxol cells to 23.8% at 8 μM. This result further confirms the utility of cajanol in reversing drug resistance by inhibiting the expression of P-gp, thus reducing the efflux of paclitaxel.

### Cajanol Regulates MDR *Via* the Akt/NF-κB/P-gp Signaling Pathway in A2780/Taxol Cells

**FIGURE 4:**
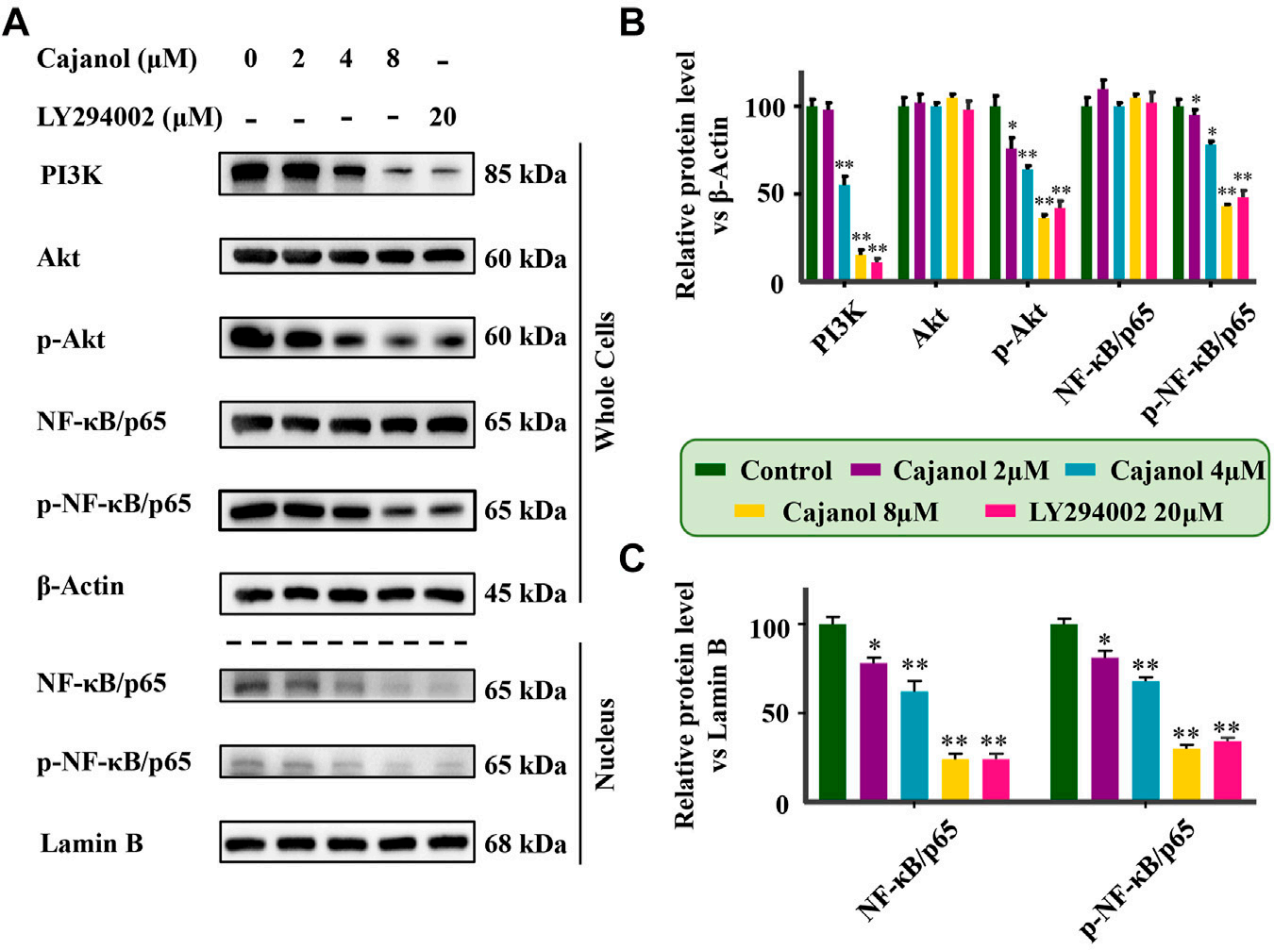
Inhibition of cajanol on PI3K/Akt/NF-κB pathway in A2780/Taxol cells. **(A)** Expression of PI3k, Akt, P-Akt, NF-κB/p65 (whole cells and nucleus) and p-F-κB/p65 (whole cells and nucleus) in A2780/Taxol cells after treatment with cajanol and LY294002. **(B,C)** Expression was normalized with *ß*-actin or lamin B, respectively. Each column shows the mean ± S.D. *p < 0.05, **p < 0.01 *vs*. control group.

The above results show that the expression of *ABCB1* and P-gp in A2780/Taxol cells is inhibited by cajanol, suggesting that cajanol could inhibit P-gp transcription and/or translation. Since Akt and NF-κB signal transduction are highly correlated with P-gp expression, it is suggested that cajanol may regulate P-gp through the Akt/NF-κB/P-gp signaling pathway. The expression of each of PI3K, Akt and phosphorylated Akt in A2780/Taxol cells was detected by western blot. Notably, cajanol reduced PI3k and *p*-Akt in A2780/Taxol cells, but did not significantly affect the expression of Akt itself (Figures 4A,B). Further, we examined the effect of cajanol on the expression of NF-κB/p65 and phosphorylated NF-κB/p65 in cells and in the nucleus. Similar to Akt and P-Akt, down-regulation of p-NF-κB/p65 was observed in 2780/Taxol cells treated with cajanol. The expression levels of NF-κB/p65 and p-NF-κB/p65 in the nucleus were down-regulated with the increase of cajanol concentration. To verify the effect of inhibiting the PI3K/Akt/NF-κB pathway on P-gp protein expression, the PI3K inhibitor LY294002 was used to treat A2780/Taxol cells. Treatment with LY294002 had effects on the expression of Akt, *p*-Akt8, NF-κB/p65, p-NF-κB/p65 and P-gp similar to those found after exposure to 8 μM cajanol. These results fully demonstrate that cajanol can inhibit PI3K and phosphorylation of Akt in A2780/Taxol cells, thereby preventing the phosphorylation and nuclear translocation of NF-κB/p65, and ultimately inhibiting the transcription and translation of P-gp.

### Cajanol Restores Taxol-Resistant Metastatic Tumor Sensitivity in Mice

**FIGURE 5:**
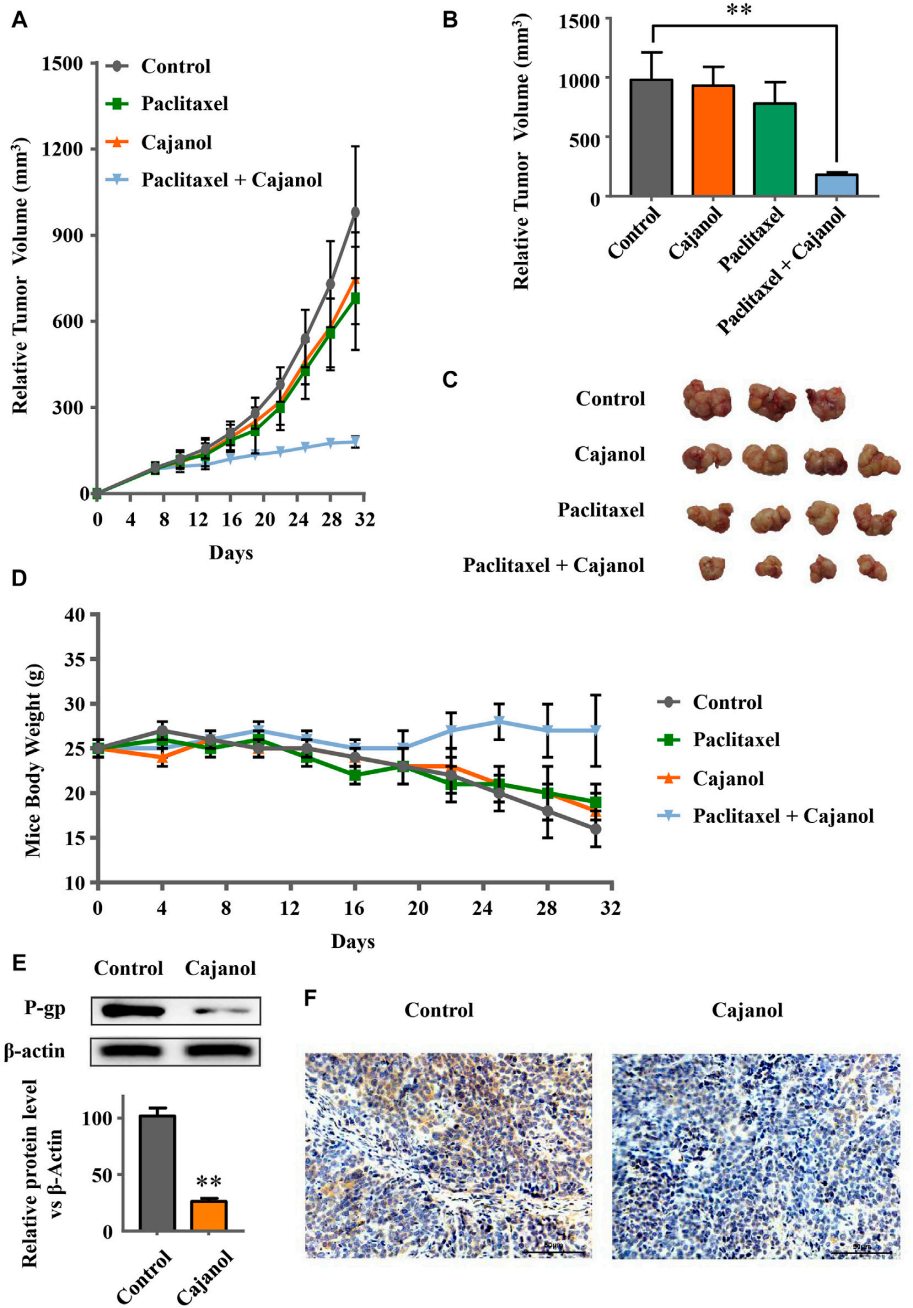
Sensitization of mice to paclitaxel by cajanol. Healthy six-week-old female mice were selected and inoculated with A2780/Taxol cells to form solid tumors. After solid tumor formation, the agents were administered once a week. Changes in the tumor volume and body weight were observed during the treatment. **(A)** Changes in the tumor volume during treatment. **(B)** Tumor growth of the mice in each group after 24 days of treatment. *p < 0.05 *vs* control group. **(C)** Tumor images of the mice after 24 days of treatment. (d) Changes in the body weight of the mice during treatment. **(E,F)** Effect of cajanol on P-gp expression in tumor tissue as determined by western blot assay and immunohistochemistry. *p < 0.05 *vs*. control group.

To verify whether cajanol can restore Taxol sensitivity in mice, we established a BABL/c nude mouse tumor model using A2780/Taxol cells with the following four groups: control, cajanol, paclitaxel and cajanol + paclitaxel, each group consisted of 4 nude mice that had been successfully modeled. After treatment for 24 days, the tumor volumes of the mice in the combined group were significantly smaller than those of the other three groups (Figures 5A–C); the final tumor volume of the cajanol + paclitaxel group was 182.4 ± 20.4 mm^3^, while tumors in the paclitaxel, cajanol and control groups had volumes of 758 ± 154 mm^3^, 680 ± 176 mm^3^ and 981 ± 215 respectively. After treatment, only the mice in the combined treatment group could maintain a body weight of approximately 25 g, while the mice in the other three groups weighed less than 20 g (Figure 5D). These results show that cajanol (2 mM/kg) combined with paclitaxel (0.5 mM/kg) *in vivo* has a significant inhibitory effect on paclitaxel-resistant cancer.

We also measured the expressions of *ABCB1* mRNA and of P-gp protein in the tumor tissues. Western blots showed that cajanol inhibits the expression of P-gp in tumor tissues, similar to the results *in vitro* (Figure 5E). The immunohistochemistry results confirm that expression of P-gp in the cajanol treatment group was significantly inhibited (Figure 5F).

## Discussion

For patients with advanced ovarian cancer, paclitaxel-based chemotherapy is currently the mainstay of treatment. However, drug resistance usually emergences, leading to treatment failure and tumor recurrence. Therefore there is a need for more research and development of new therapeutic drugs that can reverse this resistance.

It has been found that more than 90% of cancer-related deaths are due to MDR, and the main cause of MDR is the overexpression of the ABC transporters in tumors (Goldman, 2003). Among these, multidrug resistance gene *ABCB1* is considered to be one of the most important mechanisms that cause paclitaxel resistance. Overexpression of *ABCB1* leads to the overexpression of membrane P-gp, with a consequent reduction in the concentration of intracellular paclitaxel, reducing the inhibitory effect of paclitaxel on tumor cells (Schondorf et al., 2002; Lukyanova, 2010; Shen et al., 2016). The change in microtubule structure is another reason for paclitaxel resistance; the increased expression of α-tubulin and *ß*-III-tubulin will disrupt the internal stability of microtubules and reduce the efficacy of paclitaxel (Li J. et al., 2009; Kobayashi et al., 2011). In addition, matrix metalloproteinase (MMP-9) promotes tumor angiogenesis by increasing the expression of vascular endothelial growth factor (VEGFA) and its receptor (VEGFR) (Bergers et al., 2000). Abnormal expression of VEGF may be related to paclitaxel resistance. Tumor tissues release VEGF through up-regulation of P-gp (Li L. et al., 2009; Akiyama et al., 2012). In this work, we found that cajanol can inhibit the expression of *ABCB1* gene and P-gp protein, but has no effect on the expression of *VEGF*, *MMP-9*, α-tubulin or βIII-tubulin. Combined with the results of flow cytometry, we confirmed that cajanol can reduce the efflux of paclitaxel from A2780/Taxol cells by modulating the expression of P-gp protein. In addition to P-gp, the MDR of some tumor cells is caused by MDR-associated protein (MRP) (Cole et al., 1992; Sodani et al., 2012) or lung resistance-related protein (LRP) (De Figueiredo-Pontes et al., 2008). As shown in the results, the expression levels of these three proteins were low in A2780/Taxol cells, and there was no significant change after cajanol treatment.

**FIGURE 6:**
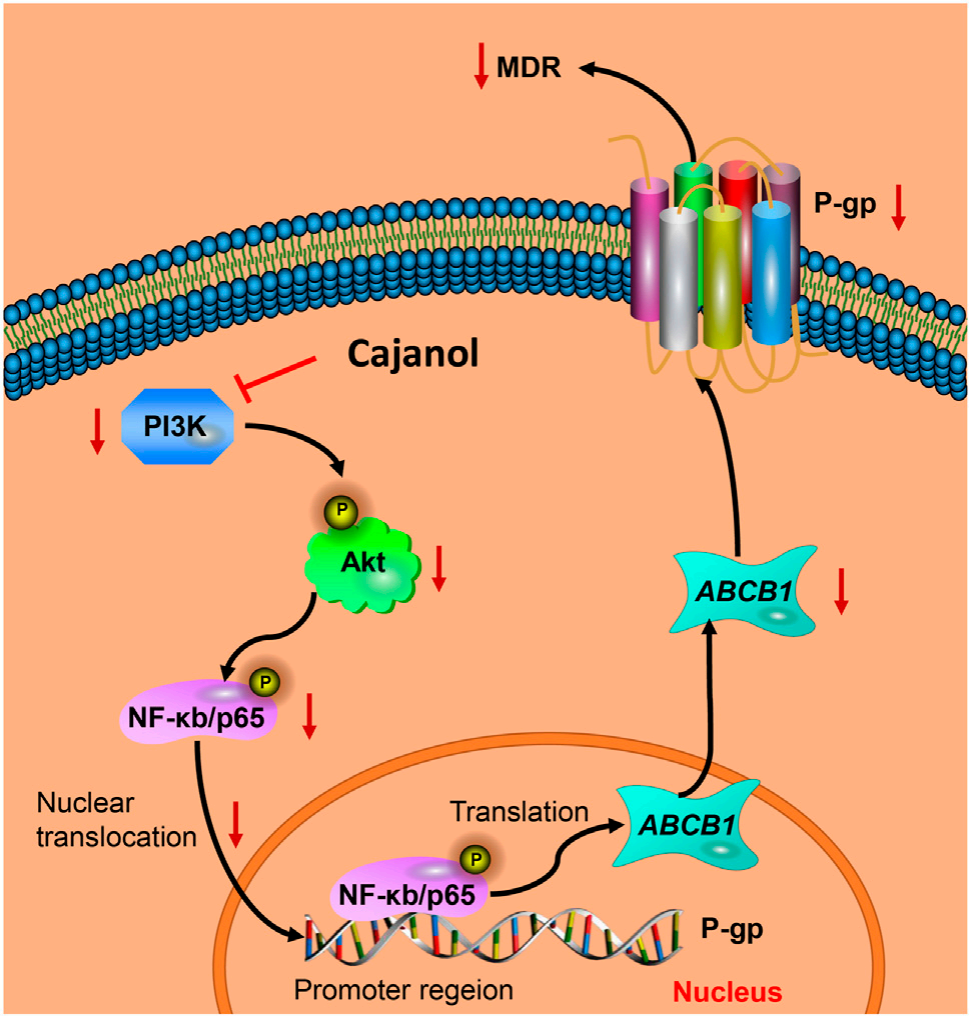
Cajanol treatment down-regulates P-gp expression via inhibition of the PI3K/Akt/NF-κB signaling pathway. Cajanol inhibits the phosphorylation of IκB and the translocation of NF-κB from the cytoplasm to the nucleus by inhibiting the expression of PI3K and the phosphorylation of Akt, and ultimately inhibits the transcription and translation of the P-gp protein.

Studies have shown that the PI3K/Akt signaling pathway is closely related to MDR, and the PI3K/Akt pathway activates the NF-κB system, which may lead to an increase in MDR1 transcription (Xi et al., 2016). When the PI3K/Akt pathway is activated, the Akt protein is phosphorylated, leading to the phosphorylation of downstream IκB-α and its dissociation from NF-κB. After nuclear translocation of NF-κB and binding to its recognition site, the promoter of the MDR1 gene is activated and gene expression is induced (Guo et al., 2016; Wu et al., 2016). Blocking the PI3K/Akt pathway can lead to down-regulation of MDR1/P-gp protein expression, thereby reversing the MDR. The results of this study show that cajanol can inhibit the expression of PI3K and the phosphorylation of Akt, thereby inhibiting the phosphorylation of IκB. It also prevents the translocation of NF-κB from the cytoplasm to the nucleus. Therefore, cajanol can inhibit P-gp expression through the PI3K/Akt/NF-κB pathway (Figure 6).

Researchers have been developing and synthesizing P-gp inhibitors to reverse MDR for the last 30 years. Verapamil, nifedipine, quinidine and cyclosporine A are substrates of P-gp that have been developed as first generation inhibitors, but they have unacceptable side effects. The second generation inhibitors, including dexverapamil and PSC833, were modified on the basis of the first-generation drugs to reduce toxicity (Boesch et al., 1991; Martin et al., 1999). These inhibitors are substrates of cytochrome P450, and they interfere with the pharmacokinetics of chemotherapeutic agents. No synthetic inhibitors have yet been approved for clinical applications, prompting researchers to explore new reversal agents from natural products. It has been reported that ferulic acid can down-regulate P-gp expression through NF-κB (Muthusamy et al., 2019). In this study, 8 μM cajanol significantly restored the sensitivity of A2780/Taxol cells to paclitaxel, and 2 mM/kg cajanol and 0.5 mM/kg paclitaxel significantly inhibited the growth of metastatic tumors in mice. The results of this study will provide new ideas for the development of effective inhibitors of MDR in ovarian cancer.

## Conclusion

In this work, cajanol inhibited phosphorylation and nuclear ectopia of NF-κB by inhibiting PI3K expression and Akt phosphorylation, thereby reducing transcription and translation of P-gp protein, and ultimately reducing MDR induced by paclitaxel efflux. This report may contribute to further explore the clinical application of cajanol in the treatment of ovarian cancer.

## Data Availability

The original contributions presented in the study are included in the article/Supplementary Material, further inquiries can be directed to the corresponding authors.
