# Supplementary material for: Cajanol Sensitizes A2780/Taxol Cells to Paclitaxel by Inhibiting the PI3K/Akt/NF-κB Signaling Pathway
Source: Front Pharmacol. 2021 Dec 8;12:783317. doi: 10.3389/fphar.2021.783317 (PMC8694871; doi:10.3389/fphar.2021.783317)
Supplement: Supplementary file 1 [file DataSheet1.DOCX]

**Supplementary Files**


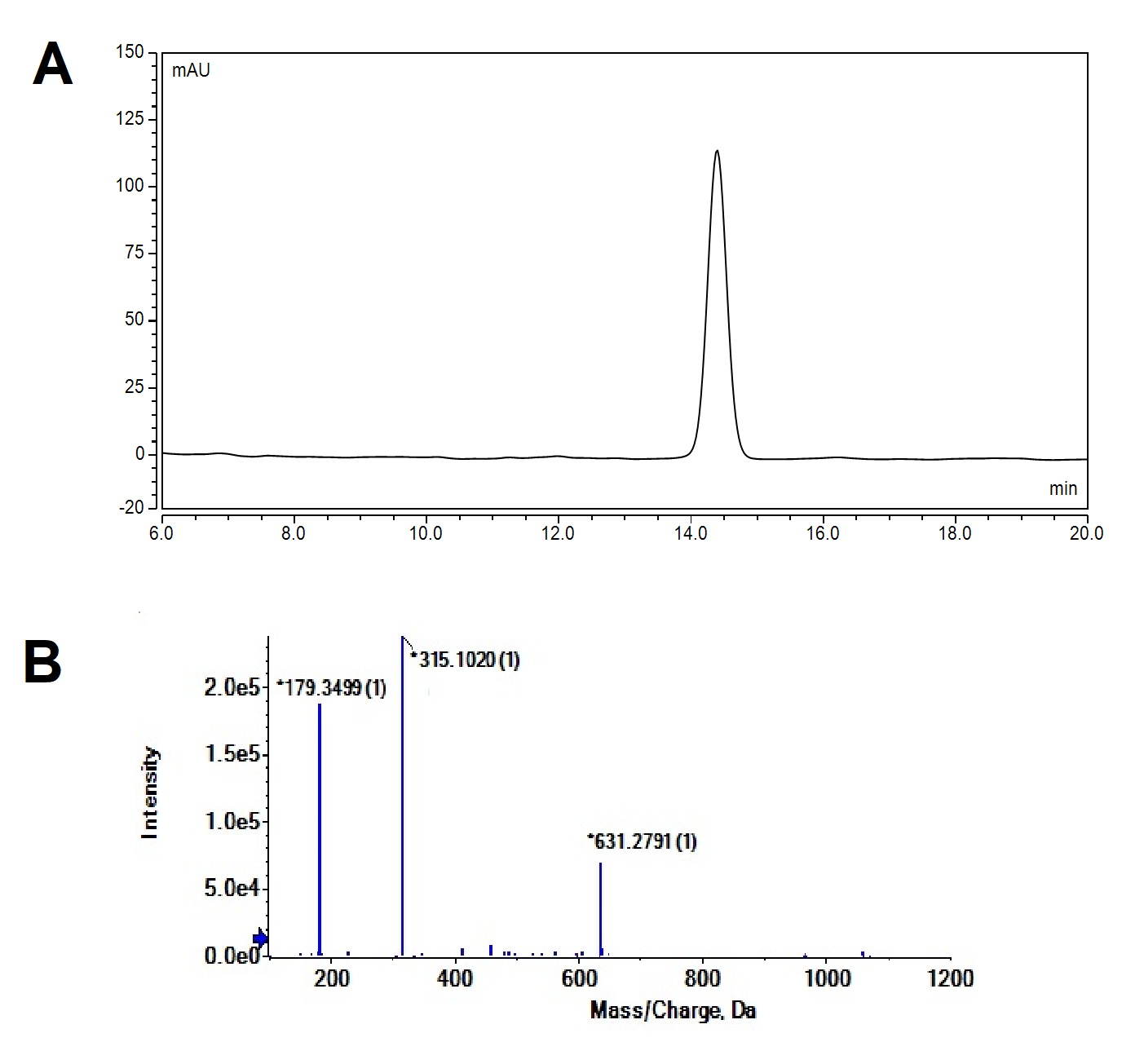


**Figure S1**. HPLC and mass spectrometry results of cajanol. **A** HPLC chromatogram of cajanol after separation and purification. **B** Secondary mass spectrometry of cajanol under negative ions mode.

**Table S1.** Fluorescent quantitative PCR primers used in this research.

| Gene | Primer direction | Primer sequence (5’→3’) |
| --- | --- | --- |
| *β-actin* | Sense | GTGGGGCGCCCAGGCACCT |
|  | Antisense | CTTCCTTAATGTCACGCACGATTG |
| *ABCB1* | Sense | GGATGAAGATGTGCCTCTGGT |
|  | Antisense | CTCCGGCTTTGCCAAATGTGA |
| *VEGF* | Sense | AGGAGTACCCCGACGAGATAGA |
|  | Antisense | CACATCTGCTGTGCTGTAGGAA |
| *βIII–tubulin* | Sense | CCCAACAACGTCAAGGTAGCC |
|  | Antisense | GATTCCTCGTCATCATCTTCATACA |
| *MMP-9* | Sense | AAAGACGACATAGACGGCATCC |
|  | Antisense | TCAGAAGAGCCCGCAGTAGG |
